# Key Sectors in the Economy of Saudi Arabia

**DOI:** 10.3389/fpubh.2021.696758

**Published:** 2021-07-27

**Authors:** Said K. M. Brika, Brahim Adli, Khalil Chergui

**Affiliations:** ^1^Department of Administrative Sciences, Community College, University of Bisha, Bisha, Saudi Arabia; ^2^Department of Economics, University of Oum El Bouaghi, Oum El Bouaghi, Algeria

**Keywords:** key sectors, backlink coefficients, Saudi Arabian economy, economic diversification, input-output model

## Abstract

This study shows the key sector for the economy of Saudi Arabia based on input-output model analyses. They derived the analyses from the economy of the Kingdom of Saudi Arabia (KSA) using 35 economic sectors. We found that four leading sectors exceeded the values of the linkage coefficients with a value of 1, represented by both chemicals and pharmaceutical products, namely, manufacturing basic metals (S13), transportation and storage (S24), and other business sector services (S31). According to the unbalanced growth theory, more attention is paid to these sectors that are the primary engine for the rest of the sectors and their growth. The results obtained are beneficial for success of the economic policy of Saudi Arabia. By observing the different influences, it is possible to identify the policies expected to have more significant indirect impacts on other sectors in Saudi Arabia and are likely to develop a prudent economic policy. Given the economic dependence on oil, it is also essential to be acquainted with the different sectors that are probable to have an overall effect on the economy for strategic and operationally effective analysis that can help.

## Introduction

This study aims to identify the key economic sectors of Saudi Arabia. A key sector analysis explores interdependent economic activities in the targeted sector. Sometimes, an estimate of interdependence is rendered using backward or forward relations. Backward linkages focus on dependencies and interdependence between different economic activities, while forward links refer to links within diverse economic sectors, e.g., Beyers (1), Hewings (2), McGilvray (3), and Rao and Harmston (4).

The general problem of recognition of key sector is its utility in the implementation of economic growth policy. According to Hirschman (5), McGilvray (3), and Veselovsky et al. (6), investing in sectors with a large degree of backward and forward linkages promotes economic growth opportunities.

Although it is essential to identify key sectors for growth planning, not commonly used in the Gulf countries, most of the debate and studies focused on developed countries. Researching the key sectors requires an enormous volume of data for decades, reducing policymakers the interests of policymakers in countries where data and financing are insufficient (7–9). Therefore, a research study in key economic sectors remains essential for decision-makers and for achieving sustainable development for each country.

As a member of the Group of Twenty (G20), the Kingdom of Saudi Arabia (KSA) has one of the strongest economies in the world; according to the Global Competitiveness Yearbook, the KSA possesses 18% of the proven oil reserves of the world, making it third among the G20 countries and the seventh in the world.

According to the International Institute for Administration, in 2019, 140 countries worldwide have varying competitiveness levels based on how well they use their available capital. The strategies to expand non-oil income streams planned to fit with Saudi Arabia strategic framework identified as “Visions Beyond Oil in Saudi Arabia” (10), toward 3.3% in 2020, as expected by these changes, would increase the development of gross domestic product (GDP) from 1.8 to 2.1% in 2020 to 2.1% of the policies proposed in 2020.

The goal is to move to the first position in global market reform and the global vanguard by 2022 and function as the most efficient country globally across 190 countries.

As per the Global Competitiveness Report 2019, Saudi Arabia was the most stable compared to the values of other countries placed on macroeconomic stability, including the inflation rates and debts. The Global Competitiveness Yearbook 2020 report showed that the ranking of the KSA has improved in three main axes: the economic performance axis, the business efficiency axis, and the infrastructure axis. As the report showed, Saudi Arabia is the only country that has made exceptional progress at the Middle East and the Arab Gulf level, according to its classification and according to the reports of the indicators. It is ranked eighth among the G20 countries.

The strategic approach of the Saudi Arabia Vision 2030 relies on diversifying non-oil income sources, unlike in the past, where reforms led to remarkable economic growth. Then, Saudi Arabia has also made progress in manufacturing, transport, logistics, renewable energy production, tourism, and mineral, and beliefs in comparative advantage compared to the development prospects of the country in each sector.

The selection of growth areas influences foreign and domestic investment in the core economic sectors that often dictates and channels the flow of economic incentives to various secondary industries, which results in widespread effects across the Kingdom. To date, these industries have been known as “Vision 2030” due to the ongoing strategic growth process. Identifying the key sectors of the economy of Saudi Arabia helps the Kingdom build long-term growth.

## Literature Review

All countries plan to diversify their economies to reduce financial shocks (11, 12). They are not subject to domestic and international fluctuations, especially those relying heavily on hydrocarbons, including Saudi Arabia. The hydrocarbon sector is greatly affected by demand and characterized by instability (13). Producers cannot control fuel prices in these circumstances, and this is what the world exposed to in the years 1997, 2001, 2008, 2014, and 2019–2020 after the epidemic that struck the whole world (14, 15). Therefore, the price of oil fell to its lowest level two decades or more ago. Consequently, it has become necessary to work on developing the rest of the sectors. Of course, it is not easy to develop all sectors at once (16). Therefore, priorities given to specific sectors that are the engine of the economy as a whole.

What did the unbalanced growth theory suggest? Hirschman and his supporters believe that this strategy is the best, especially for developing countries that lack financial resources, especially in crisis times, and suffer from a lack of experience and competence.

Looking at experiences, we find much evidence supporting the idea of unbalanced growth, including the textile sector in Britain and the railway sector in America in the Nineteenth century, the electricity sector in Russia, the food production sector in Denmark in the 20s, and the heavy industry sector in the 30s and 40s in the Twentieth century (17), and the chemical and electronic industries sector in Western Europe in the second half of the Twentieth century (18).

Therefore, the economic and standard literature studies developed several criteria to identify the leading sectors that can be relied upon and prioritize attracting the rest of the sectors, so the idea of interlocking factors calculated through the tables of inputs and outputs appeared.

The importance of this study stems from the nature of the economy of Saudi Arabia, which is linked to oil prices. It is necessary to activate other productive sectors, mainly focusing on the leading sectors, to give a strong impetus to the economy.

This study contributes to present the methodology and method of selecting the pioneering sectors through exposure to the concept of economic entanglement and its measurement. Hence, the analysis of the level of overlap between the economic sectors in Saudi Arabia in 2018 and the identification of the sectors capable of deepening the level of front and back economic entanglement in Saudi Arabia are to be taken as growth poles of priorities and development plans.

## Data

As a result, the key sectors with broad impacts have more widespread forward linkages and backward linkages above the value of 1.

Rueda-Cantuche et al. (19) provide a helpful way of visualizing the different results obtained through the key sector study (Table 1). The key sectors in Table 1 have broadly scattered backward linkages and forward linkages in the residual of the economy. Then, dynamic down the table, we identify sectors with less broadly distributed backward linkage consequences on the economy residue and sectors with no to minimal total backward effects at the bottom. Going right, we identify the sectors that have widely distributed effects of forward linkages, followed by the sectors that do not have effects of backward linkages.

**Table 1:** Hirschman/Rasmussen backward and forward linkages for the Arabia Saudi sector 2018.

**Sector**	**Backward linkage**	**Forward linkage**
S1: Agriculture, forestry, and fishing	0.714725	0.858296
S2: Mining and extraction of energy-producing products	0.615522	1.911171
S3: Mining and quarrying of non-energy-producing products	0.685589	0.895072
S4: Mining support service activities	0.688509	0.595693
S5: Food products, beverages, and tobacco	1.113257	0.813843
S6: Textiles, wearing apparel, leather, and related products	1.079993	0.866654
S7: Wood and of products of wood and cork (except furniture)	1.115989	0.757090
S8: Paper products and printing	1.084984	0.821150
S9: Coke and refined petroleum product	0.948066	1.421207
S10: Chemicals and pharmaceutical products	1.074962	1.824594
S11: Rubber and plastics products	1.104282	0.920938
S12: Other non-metallic mineral products	1.109158	0.676431
S13: Manufacture of basic metals	1.148045	2.175638
S14: Fabricated metal products, except machinery, and equipment	1.121406	0.672378
S15: Computer, electronic, and optical products	1.207329	0.637164
S16: Electrical equipment	1.219331	0.642121
S17: Machinery and equipment. e.c.	1.180979	0.621332
S18: Motor vehicles, trailers, and semi-trailers	1.104373	0.625947
S19: Other transport equipment	1.267485	0.610023
S20: Other manufacturing; repair, and installation of machinery and equipment	1.204695	0.656171
S21: Electricity, gas, water supply, sewerage, waste, and remediation services	0.981661	0.915717
S22: Construction	1.181569	0.717147
S23: Wholesale and retail trade; repair of motor vehicles	0.834946	2.664873
S24: Transportation and storage	1.072423	1.410599
S25: Accommodation and food services	0.959982	0.636167
S26: Publishing, audiovisual, and broadcasting activities	1.062945	0.601922
S27: Telecommunications	0.946771	1.229808
S28: IT and other information services	0.964516	1.003954
S29: Financial and insurance activities	0.703848	1.478544
S30: Real estate activities	0.677244	0.814451
S31: Other business sector services	1.263438	1.755292
S32: Public administration and defense; compulsory social security	0.690689	0.592983
S33: Education	0.827141	0.594016
S34: Human health and social work	1.080490	0.885178
S35: Arts, entertainment, recreation, and other service activities	0.963661	0.696435

These determinations, in particular, are the most exciting part of sectors with higher- or lower-than-average linkages and diffusion. Also, sectors with higher-than-average linkages may have comparatively limited effects than their peers in other, less trade-dependent countries in a heavily export economy. Much of the linkages are foreign trade rather than domestic businesses.

The data included the median consumption matrix and GDP column for 35 key sectors in Saudi Arabia in 2018, which we obtained from the Organization for Economic Co-operation and Development (OECD.STAT**)**.

## Methodology

The key sectors are defined by measuring the back linkages and forward linkages identified with the study of Rasmussen (20), the study of Hirschman (5), and inverse feedback of Leontief.

With main contributions from authors (21–23), the input-output table of backlinks can be determined using the standard Leontief inverse (24). While we can identify forward links using the Leontief inverse (25), there is some discussion among professionals about the approach we will use to forward linkages (26). We follow the approach of the famous Leontief (24) input-output model, and below we present the formula:

(1) $$\begin{array}{l} x=Ax+f \end{array}$$

where *x* is the vector of gross output, *f* is the vector of final demand, and *A* = (*a*_*ij*_) is the matrix of direct inputs, *a*_*ij*_ into the set of *n* production sectors, with properties:

$$\begin{array}{l} a_{ij}\geq 0,\;i,j=1,2,...,n \end{array}$$

(2) $$\begin{array}{l} \overset{n}{\underset{i=1}{\sum }}a_{ij}>0,\;j=1,2,...,n \end{array}$$

The equivalent form of the Leontief model is as follows:

(3) $$\begin{array}{l} x={\left(I-A\right)}^{-1}f=Bf \end{array}$$

where *i* is the identity (unit) matrix, and the matrix $B={\left(I-A\right)}^{-1}=\left[b_{ij}\right]$ is the Leontief inverse matrix [see Jensen and Hewings (27), Leontief (24), Miller et al. (28)].

In the Leontief inverse matrix, each sector is directly and indirectly related to all other sectors. For each sector *i*, these links are divided into two main types, backward linkages describing the direct and indirect economic inputs of different sectors to sector *i* and forward linkages representing direct and indirect economic inputs of sector *i* to all other sectors. The backward linkages of the sector are linked to column *i*, and forward linkages are linked to row *i* of Leontief inverse proportion. The concept of backward linkages and forward linkages and the related definition of key sectors are linked to the studies of Rasmussen (20) and Hirschman (5). The main thrust of this economic philosophy has been to identify sectors whose links are such that they have an over-average influence on the rest of the economy.

Rasmussen (20) proposed two types of indices:

### Power of Dispersion for the Backward Linkages

(4) $$BL_{j}=\frac{1}{n}\sum_{i\;=\;1}^{n}b_{ij}/\frac{1}{n^{2}}\sum_{i,j=1}^{n}b_{ij}=nB_{•j}/V$$

where $B_{•j}=\sum_{i=1}^{n}b_{ij}$ is the column multiplier of the sector *j*.

### Sensitivity of Dispersion for Forwarding Linkages

(5) $$\begin{array}{l} FL_{i}=\frac{1}{n}\overset{n}{\underset{j=1}{{\sum^{\text{​}}}^{\;}}}b_{ij}/\frac{1}{n^{2}}\overset{n}{\underset{i,j=1}{{\sum^{\text{​}}}^{\;}}}b_{ij}=nB_{i•}/V \end{array}$$

where *V* is the intensity (*the volume*) of the Leontief inverse *B*:

$V=\sum_{i,j}b_{ij}.$ Moreover, $B_{i•}=\sum_{j=1}^{n}b_{ij}$ is the row multiplier of the sector *i*. Moreover, all backward linkages and the sum of all forward linkages are equal to *n*, and the average linkage is equal to 1.

The usual interpretation is that the Hirschman-Rasmussen backward linkage index *BL*_*j*_ > 1 indicates that the corresponding column multiplier *B*_•*j*_ is larger than the average column multiplier.

Therefore, a unit change in final demand in sector *j* will create an above-average increase in the economy. Similarly, for the forward linkage index *FL*_*i*_ > 1, the corresponding row multiplier *FL*_*i*_ is larger than the average row multiplier. Thus, a unit change in the final demand of all sectors (including sector *i*) would create an above-average increase in sector *i*.

Using these hierarchies of backward and forward linkages, we can divide sectors into four groups:

Sector *i* is considered as a *key sector* if *BL*_*i*_ > 1 and *FL*_*i*_ > 1;
Sector *i* is considered as *a backward linkage-oriented sector* if *BL*_*i*_ > 1 and *FL*_*i*_ < 1; and
Sector *i is* defined as *a forward linkage-oriented sector* if *BL*_*i*_ < 1 and *FL*_*i*_ > 1.

I, with both backward and forward linkages <1, is considered as a *weakly linkage-oriented sector*.

A graphical illustration of the above concepts can suggest where each sector *i* represented the point *I*, with coordinates (*BL*_*i*_, *FL*_*i*_).

## Results and Discussion

Table 1 shows the results of the key sector analyses as shown below. If one looks at the weighted studies showing the current economic importance of Saudi Arabia in various sectors, some scenarios expect very encouraging surprises. Chemicals and pharmaceutical products, manufacture of basic metals, transportation and storage, and other business sector services are critical to overall production in the economy of Saudi Arabia.

The chemicals and pharmaceutical products, manufacture of basic metals, transportation and storage, and other business sector services are essential revenue sources and are essential for the employments of firms. Manufacture of basic metals is an important sector of work income; in contrast, chemicals and pharmaceutical products, transportation and storage, and other business sector services are dominant employment sectors.

The results represent all these rather specific sectors during their relationships with different sectors of the economy. These include the renewable energy sector and its basic needs for mineral materials, the tourism sector, and the importance of environmental friendliness in protecting it.

**Figure 1:**
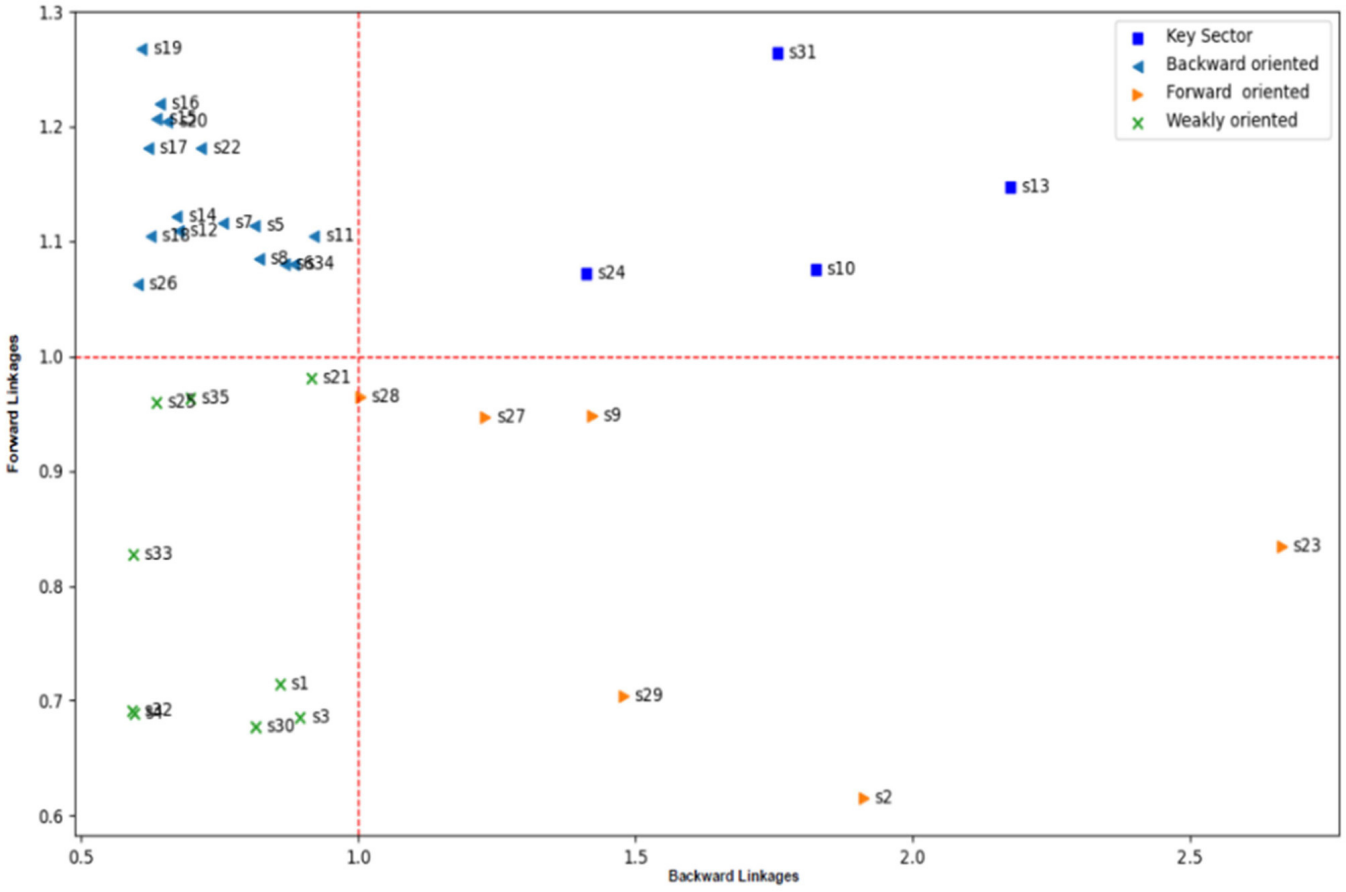
Hirschman and Rasmussen backward linkages and forward linkages for the Saudi Arabia sector 2018.

Table 1 shows the back and forward linkages for 35 economic sectors in Saudi Arabia, and the results varied between sectors and industries. There are sectors whose value exceeds 1 for both factors to represent the leading sectors. Some sectors have forward correlation coefficients more significant than representing forward-oriented sectors (the blue line exceeds a circle. The unit is according to Figure 1).

Some sectors obtained back-linking coefficients more significant than 1 to represent the backward-oriented sectors (the brown line exceeds the unit circle according to Figure 1). In contrast, the fourth group is represented by the weakly directed sectors, which obtained the coefficients. Front and rear <1.

**Figure 2:**
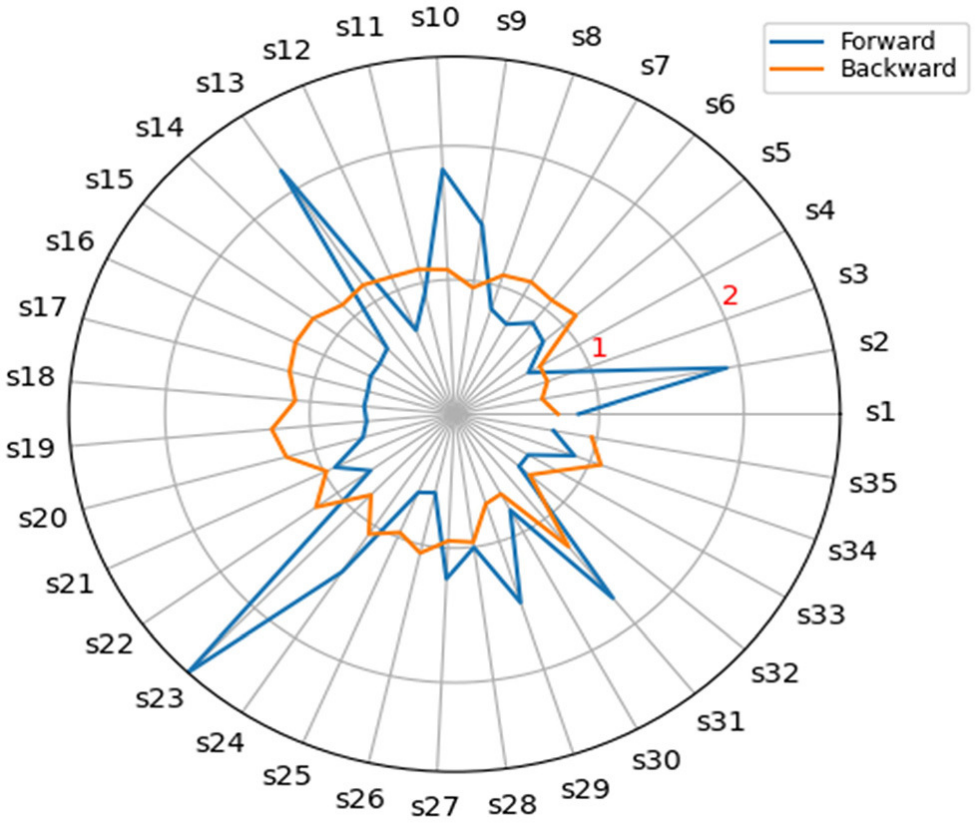
Key sectors for Saudi Arabia 2018.

The results of the study can better illustrate using the diffusion Figure 2. We found that the following sectors represent the leading sectors: the chemicals and pharmaceutical products sector (S10), the manufacture of basic metals sector (S13), the transportation and storage sector (S24), and other business sector services (S31).

Unweighted analysis, which demonstrates the influence of slight improvements in sectors on the economy as a whole, provides a more detailed view. On one hand, the forward linkage index for two different sectors is equal; this can explain the priority given the sector with the lowest value in the coefficient of variation. Hence, the comparable sectors provide their outputs relatively to many other sectors and cover the increase for every unit of the final demand. On the other hand, a high coefficient of variance value means that the sector provides its outputs to a few other sectors.

Likewise, suppose the two values of the backward linkage index for two different sectors are equal, in that case, the priority of the sector has the lowest value of the coefficient of variation, which means that the sector buys its inputs from many economic sectors, i.e., the total impact of the withdrawal is dispersed relatively equally over a considerable number of sectors.

Table 2 shows the forward coefficient of variation and backward coefficient of variation for four key sectors in Saudi Arabia. The results in the table show that the best sector among the four key sectors is S24, followed by S31, then S10, and finally S13.

**Table 2:** Forward and backward coefficient of variation for four key sectors.

**Sector**	**Backward coefficient of variation**	**Forward coefficient of variation**
S10: Chemicals and pharmaceutical products	0.044519	0.044012
S13: Manufacture of basic metals	0.045634	0.122008
S24: Transportation and storage	0.036744	0.036366
S31: Other business sector services	0.042799	0.042870

Given the nature of the economy of Saudi Arabia, it is surprising that size is key to those sectors mentioned as necessary in the unweighted test. Using the input-output scale, a significant subset of the chemicals and pharmaceutical products sector, the manufacture of basic metals sector, the transportation and storage sector, and other business sector services is critical, both backward and forward. The remaining coke and refined petroleum products, rubber and plastic products, telecommunications and information technology (IT) and other information services, and others are identified as being linked in one direction.

If one focuses on production-oriented analysis, the results point to several significant sectors where increased activity can have essential correlation effects on the economy. Among the key sectors with a broad impact are two industrial and two service sectors.

The first section gives the manufacturing sectors identified as key; the two industrial sectors are the chemicals and pharmaceutical products sector and the manufacture of basic metals sector. Those with widely sporadic impacts forwards and backwards–and depend on raw material inputs of the Saudi national economy.

In the second section, the two service sectors are transportation and storage, which are essential to the economy because they depend on many other sectors. These services are vital inputs to almost all economic activities in the Kingdom, which annually receive millions of tourists travelers to Umrah and Hadj. Then, the other services sector has different and broad effects. Especially on commercial services such as transport services, financial services represent a leading entry point for many economic activities. The findings of Alkhareif et al. (29) show that the production gap is positive on average and has financial expenditures, particularly in infrastructure, better align actual and potential. This better performance of the economy of Saudi Arabia may be due to the development of a resilient financial sector. Perhaps more unexpectedly, modern financial technology services (FinTech) constitute an essential sector that uses many inputs from other economic sectors.

## Conclusions

This study aimed to identify the key sectors of the economy of the KSA. The key sectors with less frequent elements retrospectively are those whose products support a wide range of other sectors, hence the broad potential impact. In this regard, the local key sector inputs of the economy are the chemicals and pharmaceutical products sector, the manufacture of basic metals sector, and the transportation and storage sector, and other business sector services are already a key sector. The other sectors with large-scale lagging impacts but few potential outcomes focus heavily on domestic inputs but sell primarily to export markets, mining, or tourists in Saudi Arabia. According to our study, coke and refined petroleum products, rubber and plastic products, telecommunications and IT, and other information services sectors were identified as the supporting sectors.

The mining and extraction of energy-producing products sector, wholesale and retail trade, repair of motor vehicle sectors, and financial and insurance activities sectors have been identified as policy supporters. According to our findings, there is room for higher productivity and targeting of specific sectors rather than the industrial sector on the whole range. The economic dependence of Saudi Arabia on oil and even sectors with more substantial than average links to the rest of the economy can be associated in absolute terms only stronger.

Furthermore, several of the key sectors listed here are medium-sized subsets of the economy of Saudi Arabia since several sectors are made up of many firms. It also ensures that local economies usually supply additional inputs or consume the different outputs if an industry expands exponentially. Constraints in other sectors may cause problems in practice, particularly in sectors defined as having multiplier effects, although for a limited number of others.

Besides, by observing the different influences, it is possible to identify the policies expected to have more significant indirect impacts on other sectors in Saudi Arabia and are likely to develop a prudent economic policy. Given the economic dependence on oil, it is also essential to be acquainted with the different sectors that are probable to affect the economy strategically and operationally. Therefore, effective analysis can help policymakers better understand which sectors support economic development in the Kingdom and which sector should be pushed among the important sectors of the economy and help other sectors. This information may be of paramount importance to the Vision 2030 of the Kingdom.

In light of the goals of Saudi Arabia Vision 2030, policymakers use all important economic indicators about the key sectors that provide essential information about the financial situation and contribute to reviving economic development in light of the economic conditions. In general, it is as much as possible to achieve the main goal of the Vision 2030 of the Kingdom of changing the economy to a more efficient one and diversified one. Thus, the assessment of potential production is critical, especially in moments of significant economic transformation. According to Alkhareif et al. (29), an increase in financial expenditures is necessary if the actual results are in line with the expected production. However, policies should be designed to enhance labor market efficiency through increased labor skills and participation in productive industries. It will undoubtedly help the economy of Saudi Arabia to invest in promoting a diversified economy, less dependent on natural resources. It may be the emergence of a robust financial sector in the economy of Saudi Arabia.

## Data Availability Statement

Publicly available datasets were analyzed in this study. This data can be found at: https://stats.oecd.org/Index.aspx?DataSetCode=IOTSI4_2018.

## Author Contributions

All authors listed have made a substantial, direct and intellectual contribution to the work, and approved it for publication.

## Conflict of Interest

The authors declare that the research was conducted in the absence of any commercial or financial relationships that could be construed as a potential conflict of interest.

## Publisher's Note

All claims expressed in this article are solely those of the authors and do not necessarily represent those of their affiliated organizations, or those of the publisher, the editors and the reviewers. Any product that may be evaluated in this article, or claim that may be made by its manufacturer, is not guaranteed or endorsed by the publisher.
